# The phylogeny of the Anderson's White‐bellied Rat (*Niviventer andersoni*) based on complete mitochondrial genomes

**DOI:** 10.1002/ece3.8663

**Published:** 2022-03-01

**Authors:** Shu‐Jing Liu, Fu‐Li Li, Ji‐Hua Zhou, Ji‐Zhou Lv, Zhong‐Yang Tan, Yun‐Zhi Zhang, Xing‐Yi Ge

**Affiliations:** ^1^ 12569 College of Biology Hunan Provincial Key Laboratory of Medical Virology Hunan University Changsha China; ^2^ 66359 Institute of Preventive Medicine School of Public Health Dali University Dali China; ^3^ Yunnan Provincial Key Laboratory for Zoonosis Control and Prevention Yunnan Institute of Endemic Diseases Control and Prevention Dali China; ^4^ Institute of Animal Quarantine Chinese Academy of Inspection and Quarantine Beijing China

**Keywords:** mitochondrial genome, *Niviventer andersoni*, phylogenetic analysis, taxonomy

## Abstract

The phylogenetic structure of the genus *Niviventer* has been studied based on several individual mitochondrial and nuclear genes, but the results seem to be inconsistent. In order to clarify the phylogeny of *Niviventer*, we sequenced the complete mitochondrial genome of white‐bellied rat (*Niviventer andersoni* of the family Muridae) by next‐generation sequencing. The 16,291 bp mitochondrial genome consists of 22 transfer RNA genes, 13 protein‐coding genes (PCGs), two ribosomal RNA genes, and one noncoding control region (D‐Loop). Phylogenetic analyses of the nucleotide sequences of all 13 PCGs, PCGs minus *ND6*, and the entire mitogenome sequence except for the D‐loop revealed well‐resolved topologies supporting that *N*. *andersoni* was clustered with *N*. *excelsior* forming a sister division with *N*. *confucianus*, which statistically rejected the hypothesis based on the tree of cytochrome b (*cytb*) gene that *N*. *confucianus* is sister to *N*. *fulvescens*. Our research provides the first annotated complete mitochondrial genome of *N*. *andersoni*, extending the understanding about taxonomy and mitogenomic evolution of the genus *Niviventer*.

## Graphical abstract

The complete mitochondrial genome sequence and annotation of *Niviventer andersoni*. Phylogenetic relationships among major lineages of Muridae were reconstructed using mitochondrial genomes.

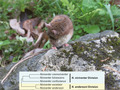

## 1 INTRODUCTION

Anderson's white‐bellied rat (*Niviventer andersoni*) belongs to genus *Niviventer*, family Muridae, and order Rodentia. *Niviventer* contains 17 recognized species with another 65 recognized as synonyms, spreading from the Himalayas and China to the Great Sunda Islands (Wilson & Reeder, 2007). All *Niviventer* species are distinguished from other murid rodents by the long, slender, flat craniums and the tail tips (Jing et al., 2007). They inhabit a variety of habitats ranging from damp forests to dry valleys. They are also in natural reservoirs or intermediate hosts for a variety of human pathogens (Keesing et al., 2010).

**FIGURE 1:**
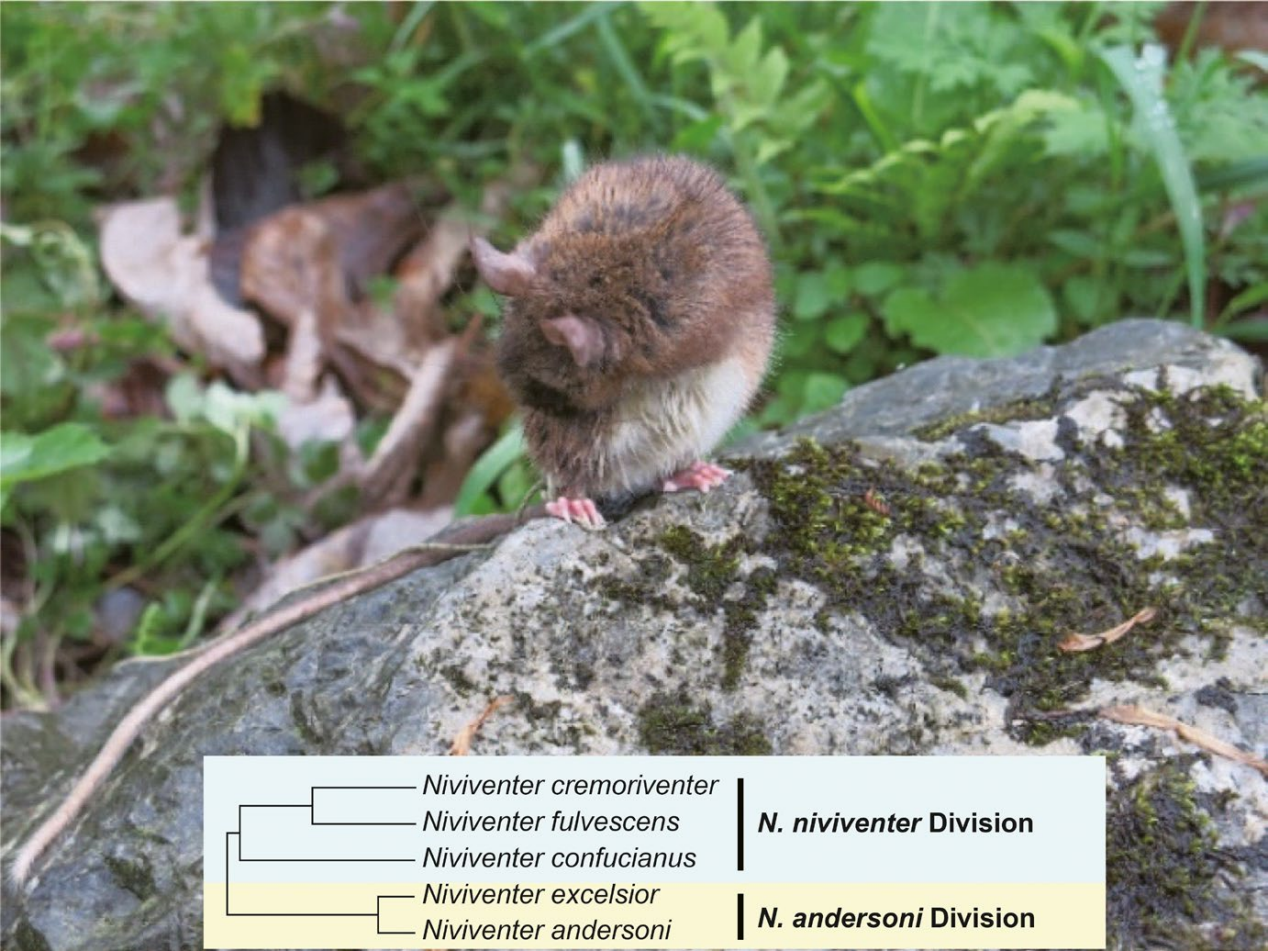
*Niviventer andersoni*. This photograph is adapted from Ge et al. (2021), Molecular Biology and Evolution, 2021 (Wilson & Reeder, 2007) under proper copyright permission. The schematic diagram of the evolutionary tree embedded in the bottom of the picture shows the *N*. *niviventer*‐Division and *N*. *andersoni*‐Division

*Niviventer andersoni* is a species endemic to China with the largest body size compared to the other congeneric species of *Niviventer* (Figure 1) (Ge et al., 2017, 2021). They live in various kinds of forests in both lowlands and mountains (Li & Yang, 2009). Fossil records showed that this species extended to the low‐altitude regions of Southeast China during the late Quaternary in Chongqing and Guizhou, suggesting they migrated southward when the climate turned colder (Bahain, 2007; Bekken et al., 2004).

The phylogenetic position of *N*. *andersoni* has not been fully determined, due to controversial phylogenetic topologies within the genus *Niviventer*. An early study proposed that the *Niviventer* could be divided into two primary groups: the *N*. *andersoni*‐Division and the *N*. *niviventer*‐Division (Musser, 1981). Phylogenetic trees based on mitochondrial cytochrome b (*cytb*) gene showed that *N*. *andersoni* and *N*. *excelsior* were clustered together and comprised the *N*. *andersoni*‐Division (Jing et al., 2007). Meanwhile, *N*. *confucianus*, together with *N*. *fulvescens* and *N*. *cremoriventer*, formed *N*. *niviventer*‐Division, a sister division to *N*. *andersoni*‐Division (Figure 1) (Ge et al., 2021; He & Jiang, 2015; Lu et al., 2015; Zhang et al., 2016). However, this classification might be inconclusive due to the limited number of gene sequences used, since each gene evolves under different evolutionary pressures and time scales (Choi & Kim, 2017). Compared to individual mitochondrial gene sequences, complete mitochondrial genome sequences can provide higher resolution and sensitivity for better revealing the evolutionary relationships among closely related species (Ladoukakis & Zouros, 2017; Wei et al., 2017).

Up to now, the mitogenomes of the four species (*N*. *andersoni*, *N*. *confucianus*, *N*. *fulvescens*, and *N*. *niviventer*) within the genus *Niviventer* have not been entirely sequenced or verified. Since complete mitochondrial genomes have been used for taxonomic and phylogenetic analyses of diverse animal groups (Kim et al., 2020; Ladoukakis & Zouros, 2017; Lavrov & Pett, 2016; Wei et al., 2017), the lack of genetic data has limited our understanding of the phylogeny of *N*. *andersoni*. In the present study, we sequenced the complete mitochondrial genome of *N*. *andersoni* and described typical features of the *N*. *andersoni* mitochondrial genome revealing its phylogenetic relationships with other white‐bellied rat species. Our findings highlight the importance of complete mitogenome information in phylogenetic analyses for rodent species.

## 2 MATERIALS AND METHODS

### 2.1 Sample collection and genomic DNA extraction

Individuals of *N*. *andersoni* were collected from Lufeng County, Yunnan province, China (*H* = 1875.43 m), in August 2018. These individuals were sacrificed and dissected for organ collection. The heart, liver, spleen, lung, kidney, and muscle were kept in the cryopreservation tubes directly. All the samples were immediately put in liquid nitrogen for short storage, then transported to the laboratory in dry ice and stored at −80°C. DNA was extracted from the muscle using mitochondrial extraction kit (Solarbio) and stored at −80°C.

### 2.2 Mitogenome sequencing, assembly, and annotation

The mitochondrial DNA was subjected to random PCR (rPCR) as previously described (Li et al., 2010). Briefly, the extracted mitochondrial DNA was first synthesized by random primer (5’‐GCC GGA GCT CTG CAG AAT TCNNNNNN‐3’). Then 2 μl of the synthetic product was used to perform the PCR in a 50‐μl reaction mixture volume containing 10 μl PCR buffer, 1 mM MgSO4, 0.2 mM each dNTP, 40 pmol universal primer (5’‐GCC GGA GCT CTG CAG AAT TC‐3’), and 1 U KOD‐Plus DNA polymerase (Toyobo, Japan). The reaction was conducted for 40 cycles of 94°C for 30 s, 54°C for 30 s, and 68°C for 2 min followed by incubation for 10 min at 68°C. The products were analyzed by agarose gel electrophoresis.

The purified rPCR products were used to construct the sequencing library and sequenced on HiSeq‐PE150 instrument (TIANGEN, Beijing, China). The raw reads were trimmed and filtered using Trimmomatic (Version 0.39) (Bolger et al., 2014). The cleaned reads were aligned to NCBI non‐redundant protein sequence database using BLASTx by DIAMOND (Buchfink et al., 2015). Mitochondrial reads were selected and de novo assembled into a complete mitochondrial genome using Geneious software package (Version 2019.1.1) (Kearse et al., 2012). Protein‐coding genes (PCGs) were annotated using the NCBI ORF Finder (https://www.ncbi.nlm.nih.gov/orffinder/) and BLASTx with the vertebrate mitochondrial genetic code. The tRNA genes were identified using the tRNAscan‐SE Search Server under the default search mode, using the vertebrate mitochondrial genetic code source (Chan & Lowe, 2019). Composition skew analysis was calculated according to the formulas: AT skew = (A–T)/(A + T) and GC skew = (G–C)/(G + C) (Perna & Kocher, 1995). Relative synonymous codon usage (RSCU) values were calculated using CodonW 1.4.2 (Wu et al., 2007). The circular mitochondrial genome map of *N*. *andersoni* was drawn using OGDRAW 1.3.1 (Greiner et al., 2019).

### 2.3 Phylogenetic analysis

Phylogenetic analysis was performed by comparing mitogenome sequences of *N*. *andersoni* with four other white‐bellied rats in *Niviventer* genus and additionally with genomes of murid rodents from the genus *Rattus* (*R*. *andamanensis*, *R*. *baluensis*, *R*. *norvegicus*, *R*. *tanezumi*, *R*. *tiomanicus*), *Mus* (*M*. *musculus*), and *Leopoldamys* (*L*. *edwardsi*, *L*. *sabanu*s) as outgroups and calibration points of the phylogeny (Table 1). Meanwhile, *cytb*, *cox1*, and *ND2*, the three genes commonly for phylogenetic analyses, were used to construct phylogenetic control trees. To further investigate the phylogenetic relationships of *N*. *andersoni*, the phylogenetic relationships were reconstructed based on the complete mitochondrial genome. The D‐loop region was excluded because of the rapid mutation rate in this region. The nucleotide sequences were aligned using ClustalX with default settings before concatenation by DAMBE (Version 7.2) (Larkin et al., 2007; Xia, 2017). Models of evolution were evaluated using corrected Aikake Information Criteria (AICc) in jModelTest 2.1.10 to determine the best nucleotide substitution model (Darriba et al., 2012). Maximum likelihood (ML) analysis of the 13 PCGs in 13 species of rodent was also performed using MEGA X (Kumar et al., 2018). The support values of the ML tree were evaluated via a bootstrap test with 1000 iterations.

**TABLE 1:** Complete mitochondrial genomes used for phylogenetic analysis in this study

Genus	Species	Common name	Gen Bank
*Leopoldamys*	*Leopoldamys edwardsi*	Edwards's long‐tailed giant rat	NC_025670.1
	*Leopoldamys sabanus*	Long‐tailed giant rat	MN964122.1
*Mus*	*Mus musculus*	House mouse	NC_005089.1
*Niviventer*	*Niviventer confucianus*	Chinese, white‐bellied rat	NC_023960.1
	*Niviventer cremoriventer*	Dark‐tailed tree rat	NC_035822.1
	*Niviventer excelsior*	Large white‐bellied rat	NC_019617.1
	*Niviventer fulvescens*	Chestnut white‐bellied rat	NC_028715.1
*Rattus*	*Rattus andamanensis*	Indochinese forest rat	NC_046686.1
	*Rattus baluensis*	Summit rat	NC_035621.1
	*Rattus norvegicus*	Norway rat	NC_001665.2
	*Rattus tanezumi*	Oriental house rat	NC_011638.1
	*Rattus tiomanicus*	Malayan field rat	MN126562.1

### 2.4 Estimation of divergence date

The 13 PCG sequences were aligned using Muscle program by codon method in MEGA. We calculated the differentiation time of species using 13 PCGs as different partitions in BEAST v1.10.4 program (Drummond et al., 2012). The substitution models and clock models (uncorrelated lognormal relaxed clock) were unlinked among partitions in BEAST. The most appropriate substitution and variant sites model of each PCG through ModelFinder program was estimated according to the Bayesian Information Criterion (BIC) method (Kalyaanamoorthy et al., 2017), the result is shown in Table S1. Referring to previous reports of divergence dates in *Rattus* genus, two priors of the most recent common ancestor (tMRCA) were used to calibrate the molecular clock (Camacho‐Sanchez & Leonard, 2020; Robins et al., 2008). The tMRCA of *Rattus* genus was set to normal distribution with mean value of 3.5 Mya and sdtev value of 0.25; the tMRCA of *Rattus baluensis* and *Rattus tiomanicus* was set to normal distribution with mean value of 0.31 Mya and sdtev value of 0.1. The Yule process speciation model was used in tree priors. We ran the Markov chain of 120 million steps and sampling every 10,000 steps. The Tracer v1.7 program was used for checking the Effective Sample Size (ESS) of each parameter and ensured that they all reached convergence (ESS > 200), and the Maximum Clade Credibility (MCC) tree was created after discarding the first 10% of states by Tree Annotator program.

## 3 RESULTS

### 3.1 Genome organization

**FIGURE 2:**
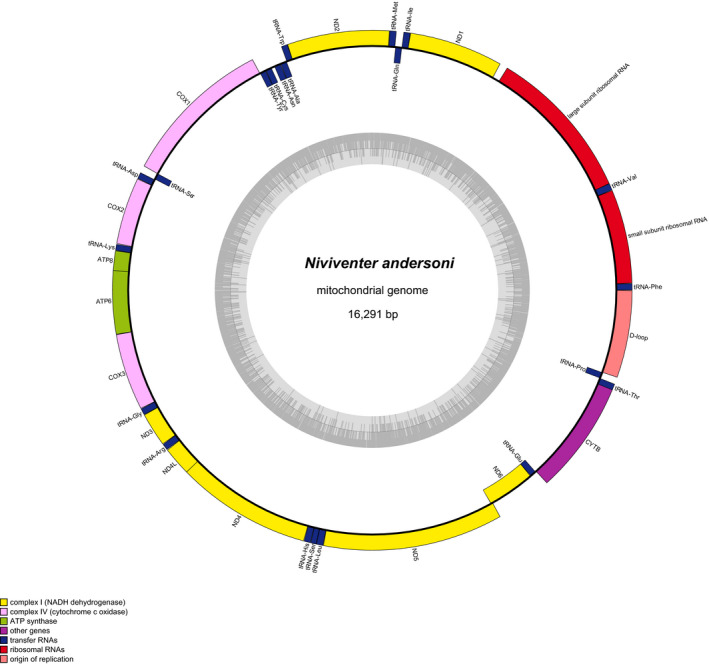
Mitochondrial genome map of *Niviventer andersoni*

From the raw reads, a total of 1,578,672 high‐quality reads were obtained and used to assemble the *N*. *andersoni* mitochondrial genome. As a result, the complete mitochondrial genome sequence of *N*. *andersoni* was deposited into NCBI with GenBank accession number MW030174. The mitogenome of *N*. *andersoni* was a circular DNA molecule with 16,291 bp in length. As shown in Figure 2, the mitogenome organization of *N*. *andersoni* was similar to those of other rodents (Boore, 1999). Thirty‐seven typical mitochondrial genes were identified, including 13 PCGs, 22 tRNA genes, and 2 rRNA genes (Table S2). Most of the genes were encoded by the heavy strand (H‐strand), while ND6 and 8 tRNAs were encoded by the light strand (L‐strand).

The total base composition of *N*. *andersoni* mitochondrial genome was estimated to be 33.7% for A, 25.8% for C, 12.1% for G, and 30.0% for T, making AT and GC percentage as 61.6% and 38.4%, respectively, indicating that the mitochondrial genome biased toward AT (Table 2). Such base composition bias has been reported to play a vital role in the replication and transcription of mitochondrial genome (Wei et al., 2010). It also showed a negative GC skew value (−0.347), indicating that C was more common than G whereas the AT skewness was positive (0.092) suggesting that A occurred more frequently than T in the *N*. *andersoni* mitochondrial genome (Table 2).

**TABLE 2:** Nucleotide composition and AT‐GC skewness of the *Niviventer andersoni* mitogenome

*Niviventer andersoni*	Size (bp)	A	G	T	C	A+T	AT skewness	GC skewness
Mitogenome	16,291	33.65	12.53	27.97	25.85	61.62	0.092	−0.347
PCGs	12,309	28.96	11.51	27.29	25.01	56.25	0.030	−0.370
tRNAs	1499	34.62	18.55	30.29	16.54	64.91	0.067	0.057
rRNAs	2524	38.19	16.48	25.24	20.09	63.43	0.204	−0.099
Control region	889	34.31	11.36	29.92	24.41	64.23	0.068	−0.365

### 3.2 Protein‐coding genes

**FIGURE 3:**
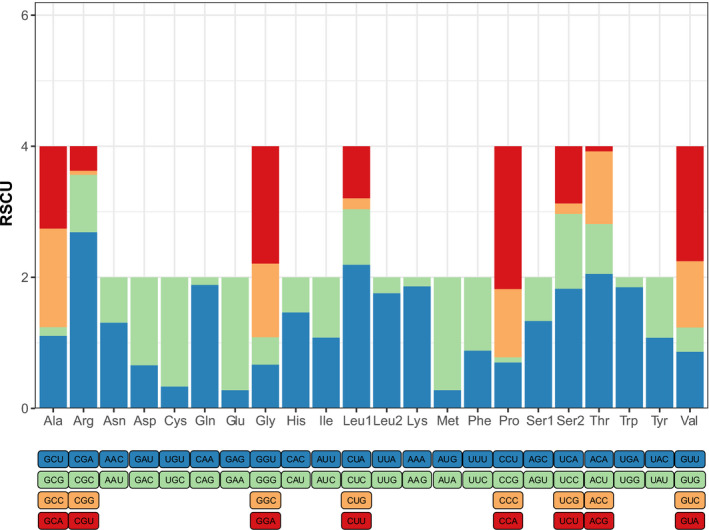
The relative synonymous codon usage (RSCU) in the mitogenome of *Niviventer andersoni*. The box below the bar chart represents all codons encoding each amino acid, and the height of the column above represents the sum of all RSCU values

The total length of the 13 PCGs was 11,420 bp, composing 70.1% of the mitogenome. The initiation codons of all PCGs in mitogenome of *N*. *andersoni* were typical ATN, except for *ND1*, which started with GTG. All PCGs of the mitogenome of *N*. *andersoni* terminated with complete (TAA) or truncated (T) stop codons, except for *ND2* which terminated with CAT (Table S2). The RSCU values of PCGs are displayed in Table S3, which also show that the PCG region has 3,805 codons. According to the RSCU analyses, CUA (L), AUU (I), and AUA (M) were the three most frequently used codons. Leucine, isoleucine, and threonine were the most frequent PCG amino acids (Figure 3). This may explain the negative GC skew and positive AT skew of PCGs.

### 3.3 Ribosomal RNA and Transfer RNA genes

The mitogenome of *N*. *andersoni* contained the typical 22 tRNA genes throughout the genome and appeared to be highly A+T biased, ranging in length from 59 bp to 75 bp. Among these tRNA genes, 8 were transcribed on the L‐strand and the remaining 14 were transcribed on the H‐strand (Table S2). All the tRNA genes exhibited a typical cloverleaf structure, except trns1 which lacked a dihydroxyuridine (DHU) arm that was simplified to a ring shape. Loss of the DHU arm is common in the mitogenomes of many mammal animals (Wolstenholme, 1992).

The two rRNA genes (lrRNA, srRNA) encoding the small and large ribosomal subunits were located between tRNA^Phe^ and tRNA^Leu^ on the L‐strand of *N*. *andersoni*. The lrRNA and srRNA genes were 1567 and 957 bp in length, respectively. The A+T content of rRNA was 63.43%, and its AT skew (0.204) and GC skew (−0.099) showed that more As and Cs were present in the rRNA than As and Gs (Table 2).

### 3.4 Phylogenetic analysis

**FIGURE 4:**
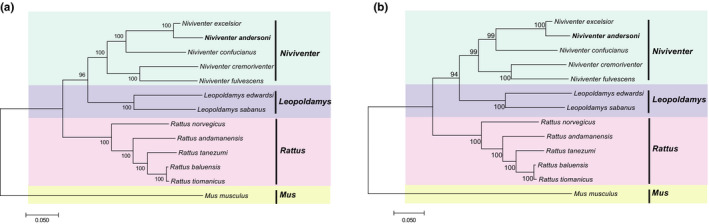
The maximum likelihood analyses of phylogenetic relationship based on (a) 13 PCGs and (b) 12 PCGs of 13 species

Based on 13 PCGs of 13 species, we obtained a phylogenetic tree by ML method with 1000 replications in which *Mus musculus* was set as the outgroup (Figure 4a). Previous research has suggested that *ND6* gene should be excluded during phylogenetic analysis due to its high heterogeneity and consistently poor phylogenetic performance (Miya & Nishida, 2000). Thus, we constructed another phylogenetic tree based on PCGs excluding *ND6* (Figure 4b). As a result, the two phylogenetic analyses were similar. When compared with other white‐bellied rat species, *N*. *andersoni* was phylogenetically closer to *N*. *excelsior* and clustered within genus *Niviventer*. For the three independent trees generated for *cytb*, *cox1*, and *ND2*, we obtained three different topological structures. The *ND2* tree showed similar topologies to the 13 PCGs combined, both showing that *N*. *andersoni* and *N*. *confucianus* were sister species. The *cox1* tree showed a cluster of *N*. *confucianus*, *N*. *cremoriventer*, and *N*. *fulvescens*. For *cytb*, *N*. *confucianus* was clustered with *M*. *musculus* (Figure S1).

**FIGURE 5:**
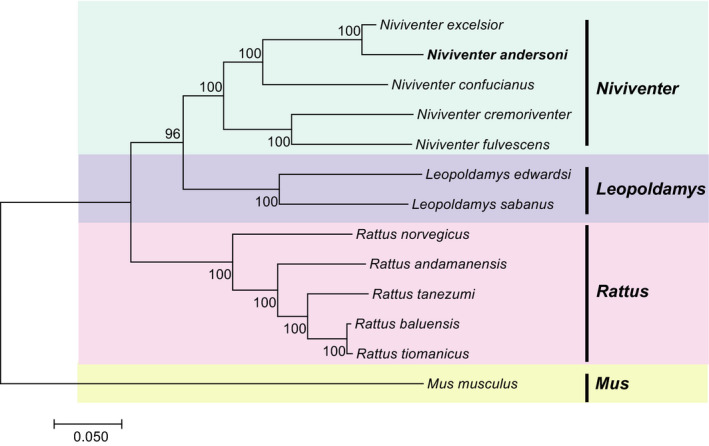
The maximum likelihood analyses of phylogenetic relationships based on complete mitochondrial genome minus the D‐loop

**FIGURE 6:**
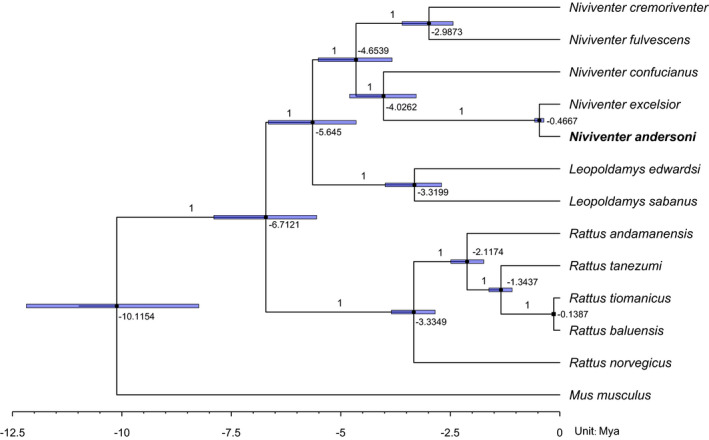
The tree with divergence time based on 13 PCGs. The value near the node indicates the age of the node, and the node shape indicates the 95% HPD range. The label on the branch represents the Bayesian posterior probability

The ML tree constructed based on the complete mitochondrial genome (except D‐loop) showed the same topologies as those PCG trees (Figure 5). In addition, we estimated that the divergence date of *Niviventer* genus was about 4.65 million years ago (Mya) with 95% HPD of 3.83 Mya ~5.52 Mya, and the tMRCA of *N*. *andersoni* and *N*. *excelsior* was about 0.47 Mya (0.37 Mya ~0.57 Mya, 95% HPD). The divergence date of *N*. *andersoni* and *N*. *excelsior* cluster from *N*. *confucianus* was about 4.03 Mya with 95% HPD of 3.28 Mya ~4.80 Mya (Figure 6).

## 4 DISCUSSION

In this study, we obtained the first annotated complete mitochondrial genome sequence of *N*. *andersoni* and conducted phylogenetic analyses based on the nucleotide sequences widely covering the mitochondrial genome, including all 13 PCGs, PCGs minus *ND6*, and the entire mitogenome sequence except for the D‐loop. The results provided a comprehensive view of the phylogenetic position of *N*. *andersoni* and the phylogeny structure of the genus *Niviventer*.

The phylogenetic topologies within the genus *Niviventer* have been studied for decades. This genus was initially divided into two groups based on *cytb* gene that were the *N*. *andersoni*‐Division including *N*. *andersoni* and *N*. *excelsior*, and the *N*. *niviventer*‐Division including *N*. *confucianus*, *N*. *fulvescens*, and *N*. *cremoriventer* (Jing et al., 2007). However, as more *Niviventer* species were found and phylogeny on several other mitochondrial and nuclear genes was analyzed, it was proposed that this genus should be classified into four groups, or species complexes. Typically, *N*. *andersoni* and *N*. *excelsior* were still classified into the same division, but *N*. *confucianus* was in a division different from *N*. *fulvescens* and *N*. *cremoriventer* (Ge et al., 2021; He & Jiang, 2015; Lu et al., 2015; Zhang et al., 2016). Although the latter classification has been accepted by most researchers recently, the phylogenetic topologies were still based on a limited number of genes only, which might lead to bias. Therefore, more genetic sequence, especially the nuclear genome, is required to further clarify it.

Our results based on the complete mitochondrial genome partially supported the latter four‐division taxa. The results suggest that *N*. *andersoni* and *N*. *excelsior* clustered together, then with *N*. *confucianus*, and these three formed a division, and *N*. *fulvescens* and *N*. *cremoriventer* formed another division. Since each gene evolves under different evolutionary pressure and time scale, it has been known that one gene tree for a population may differ from other gene trees for the same population depending on the subjective selection of the genes (Choi & Kim, 2017). Our results also suggested that the inconsistency among evolutionary trees of mitochondrial single gene could be verified by mitochondrial whole genome evolutionary analysis.

Nevertheless, the phylogenetic topologies of the genus *Niviventer* revealed by maternally inherited mitochondrial genome alone may not agree with that obtained with nuclear genome, due to incomplete lineage sorting, mitochondrial introgression, and recent hybridization (Barbosa et al., 2018; Rubinoff & Holland, 2005). The long divergence time of *N*. *andersoni*/*N*. *excelsior* and *N*. *confucianus* also indicates a potential large genetic difference between them. Thus, nuclear genomic sequences are required to draw a complete picture of the *Niviventer* taxa. Unfortunately, complete nuclear genomic sequences are not available for any *Niviventer* species. Therefore, more nuclear genes or even complete nuclear genome should be included in the analysis to provide more comprehensive view of the phylogenetic topologies in the genus *Niviventer*.

## 5 CONCLUSION

We have sequenced and annotated the complete mitochondrial genome of *N*. *andersoni* for the first time and compared it with closely related species of the family Muridae. The mitochondrial genome structural features were similar to the other species in genus *Niviventer*. In the phylogenetic analysis based on the sequences of the 13 PCGs, the PCGs excluding *ND6*, and the complete mitogenome without D‐loop, *N*. *andersoni* was consistently clustered with *N*. *excelsior*, together forming a sister group of *N*. *confucianus*. The complete mitochondrial genome of *N*. *andersoni* will extend our understanding about the evolution of the genus *Niviventer*, as well as its relationship to other murid rodents.

## CONFLICT OF INTEREST

The authors declare that there is no conflict of interests.

## AUTHOR CONTRIBUTIONS

**Shu‐Jing Liu:** Conceptualization (equal); Data curation (equal); Formal analysis (equal); Investigation (equal); Software (equal); Validation (equal); Visualization (equal); Writing – original draft (equal). **Fu‐Li Li:** Resources (equal). **Ji‐Hua Zhou:** Resources (equal). **Ji‐Zhou Lv:** Resources (equal). **Zhong‐Yang Tan:** Resources (equal). **Yun‐Zhi Zhang:** Project administration (equal); Supervision (equal); Resources (equal). **Xing‐Yi Ge:** Project administration (equal); Supervision (equal); Writing – review & editing (equal).

## Supporting information

Appendix S1

## Data Availability

The following information was supplied regarding the availability of DNA sequences: The complete mitogenome of *Niviventer andersoni* is deposited in GenBank of NCBI under accession number MW030174.
