# Supplementary material for: The phylogeny of the Anderson's White‐bellied Rat (Niviventer andersoni) based on complete mitochondrial genomes
Source: Ecol Evol. 2022 Mar 1;12(3):e8663. doi: 10.1002/ece3.8663 (PMC8890005; doi:10.1002/ece3.8663)
Supplement: Supplementary file 1 — Appendix S1 [file ECE3-12-e8663-s001.pdf]

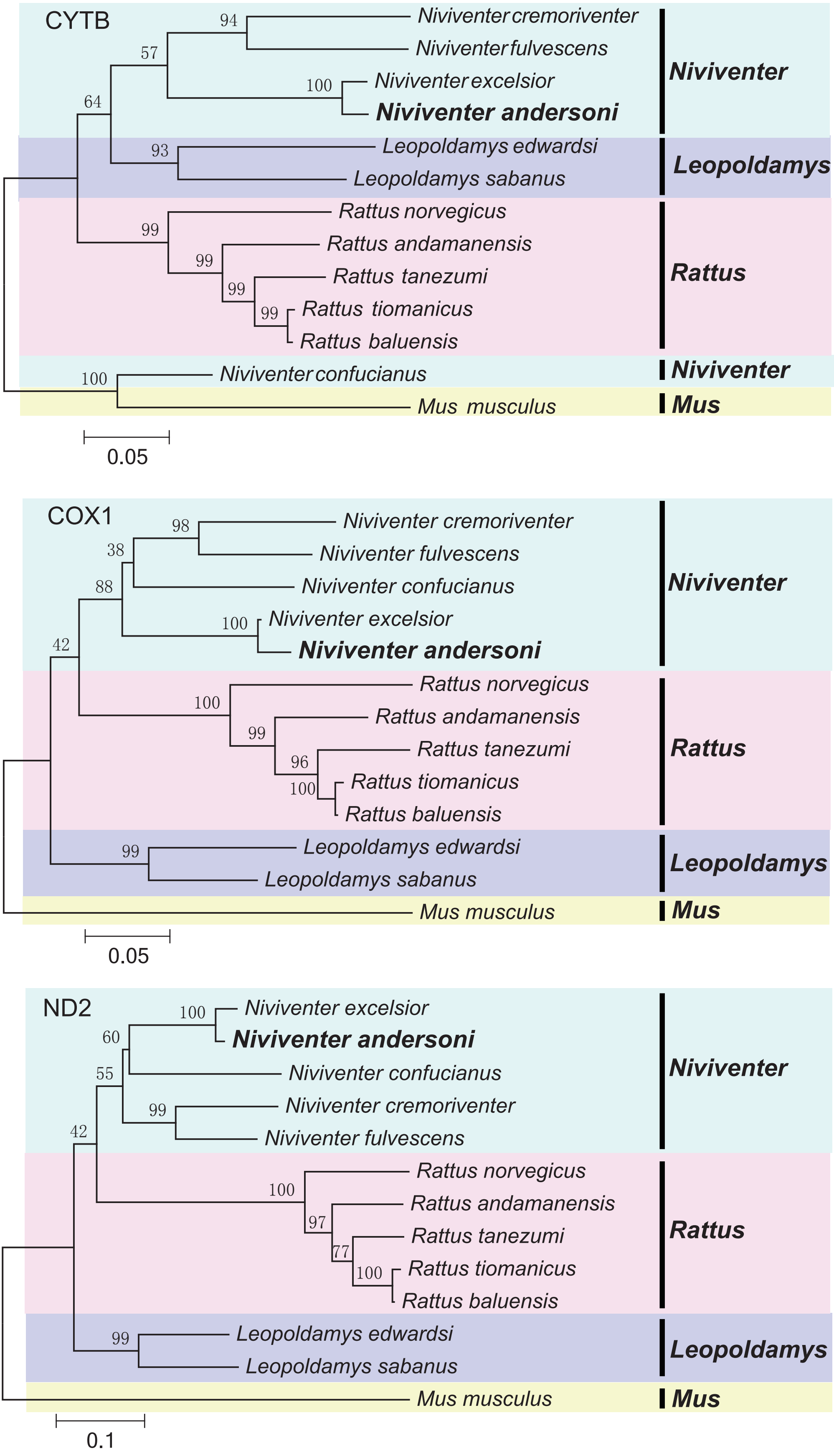

Fig. S1. The maximum likelihood analyses of phylogenetic trees based on *cytb*, *cox1*, and *ND2* genes of 13 species, respectively.

Table S1. The evolutionary model of 13 PCGs of 13 white-bellied rat species.

Gene	Evolutionary model
ATP6	TVM+F+G4
ATP8	HKY+F+G4
COX1	GTR+F+G4
COX2	TN+F+G4
COX3	GTR+F+G4
CYTB	GTR+F+G4
ND1	GTR+F+G4
ND2	HKY+F+G4
ND3	HKY+F+G4
ND4	TN+F+G4
ND4L	HKY+F+G4
ND5	GTR+F+G4
ND6	HKY+F+G4

Table S2. Characteristics of the mitochondrial genome of *Niviventer andersoni*

Gene	Start	Stop	Length(bp)	Start Codon	Stop Codon	Strand	A+T
tRNA-Phe	1	68	68			H	67.6%
s-rRNA	69	1025	957			H	62.6%
tRNA-Val	1026	1093	68			H	58.8%
l-rRNA	1094	2660	1567			H	63.9%
tRNA-Leu2	2661	2735	75			H	54.7%
ND1	2736	3690	955	GTG	T--	H	57.6%
tRNA-lle	3691	3759	69			H	72.5%
tRNA-Gln	3757	3827	71			L	62.0%
tRNA-Met	3831	3899	69			H	52.2%
ND2	3900	4935	1036	ATC	CAT	H	63.7%
tRNA-Trp	4936	5001	66			H	63.6%
tRNA-Ala	5003	5071	69			L	69.6%
tRNA-Asn	5073	5143	71			L	66.2%
tRNA-Cys	5178	5245	68			L	50.0%
tRNA-Tyr	5246	5311	66			L	54.5%
COX1	5313	6857	1545	ATG	TAA	H	59.7%
tRNA-Ser2	6855	6923	69			L	59.4%
tRNA-Asp	6927	6994	68			H	82.4%
COX2	6996	7679	684	ATG	TAA	H	59.5%
tRNA-Lys	7683	7747	65			H	66.2%
ATP8	7748	7951	204	ATG	TAA	H	64.2%
ATP6	7909	8589	681	ATG	TAA	H	62.7%
COX3	8589	9372	784	ATG	T--	H	57.4%
tRNA-Gly	9373	9440	68			H	64.7%
ND3	9441	9788	348	ATC	TAA	H	62.9%
tRNA-Arg	9790	9857	68			H	80.9%
ND4L	9860	10156	297	ATG	TAA	H	63.3%
ND4	10150	11527	1378	ATG	T--	H	62.0%
tRNA-His	11528	11595	68			H	73.5%
tRNA-Ser1	11596	11654	59			H	62.7%
tRNA-Leu1	11654	11724	71			H	66.2%
ND5	11725	13554	1830	ATA	TAA	H	61.3%
ND6	13532	14050	519	ATG	TAA	L	61.3%
tRNA-Glu	14051	14119	69			L	69.6%
CYTB	14125	15283	1159	ATG	TAA	H	58.3%
tRNA-Thr	15269	15335	67			H	68.7%
tRNA-Pro	15336	15402	67			L	62.7%
D-loop	15403	16291	889			H	64.2%

Table S3. Relative synonymous codon usage and codon numbers in *Niviventer andersoni* mitochondrial protein - coding genes

Codon	Count	RSCU	Codon	Count	RSCU	Codon	Count	RSCU	Codon	Count	RSCU
UCA(S)	115	1.83	AUG(M)	32	0.28	GGU(G)	35	0.67	UAG(*)	0	0.00
UCC(S)	72	1.14	AUA(M)	198	1.72	GGG(G)	22	0.42	UAA(*)	8	4.00
UCG(S)	10	0.16	AAC(N)	102	1.31	GGC(G)	59	1.12	AGA(*)	0	0.00
UCU(S)	55	0.87	AAU(N)	54	0.69	GGA(G)	94	1.79	AGG(*)	0	0.00
ACA(T)	159	2.05	CCU(P)	35	0.70	CAC(H)	71	1.46	GCU(A)	67	1.11
ACU(T)	59	0.76	CCG(P)	4	0.08	CAU(H)	26	0.54	GCG(A)	8	0.13
ACC(T)	86	1.11	CCC(P)	52	1.04	AUU(I)	200	1.08	GCC(A)	91	1.50
ACG(T)	6	0.08	CCA(P)	109	2.18	AUC(I)	170	0.92	GCA(A)	76	1.26
GUU(V)	35	0.86	CAA(Q)	81	1.88	AAA(K)	94	1.86	UGU(C)	5	0.33
GUG(V)	15	0.37	CAG(Q)	5	0.12	AAG(K)	7	0.14	UGC(C)	25	1.67
GUC(V)	41	1.01	CGA(R)	43	2.69	CUA(L)	251	2.19	GAU(D)	25	0.66
GUA(V)	71	1.75	CGC(R)	14	0.88	CUC(L)	97	0.85	GAC(D)	51	1.34
UGA(W)	98	1.85	CGG(R)	1	0.06	CUG(L)	19	0.17	GAG(E)	13	0.28
UGG(W)	8	0.15	CGU(R)	6	0.38	CUU(L)	91	0.80	GAA(E)	81	1.72
UAC(Y)	69	1.08	AGC(S)	38	1.33	UUA(L)	116	1.76	UUU(F)	104	0.88
UAU(Y)	59	0.92	AGU(S)	19	0.67	UUG(L)	16	0.24	UUC(F)	132	1.12
